# Cross-Sectional Analysis of Prevalence and Aetiological Factors of Dental Erosion in Turkish Children Aged 7–14 Years

**DOI:** 10.3290/j.ohpd.a45436

**Published:** 2020-10-27

**Authors:** Elif Korkmaz, Arife Kaptan

**Affiliations:** a DDS, Cumhuriyet University, Faculty of Dentistry, Department of Pediatric Dentistry, Sivas, Turkey. Data collection and analysis; writing the manuscript.; b Associate Professor, Cumhuriyet University, Faculty of Dentistry, Department of Pediatric Dentistry, Sivas, Turkey. Conception; data analysis; writing the manuscript.

**Keywords:** dental erosion, aetiological, paediatric dentistry, prevalence, O’Sullivan’s erosion index

## Abstract

**Purpose:**

This study aimed to estimate the prevalence, distribution, and the associated factors of tooth erosion in Turkish school children.

**Materials and Methods:**

A cross-sectional analysis was performed on a representative sample of 473 children (aged 7–14 years) from 11 public schools in Turkey. Parents were asked to fill out a questionnaire to collect sociodemographic data. A questionnaire was also given to the children, to collect data pertaining to personal demographic details and habits of consuming acidic foods and drinks. The O’Sullivan index was used to assess affected permanent teeth. The data were analysed using a chi-square test and multivariate logistic regression analysis.

**Results:**

Dental erosion was observed in 21.8% of the children. Lesions were most often observed in the enamel with less than half of the buccal surface affected. Erosion was found to be statistically significantly higher in older children and in those with an elevated body mass index (BMI) (p <0.05). The consumption of fruit juices, drinks with cola, orange soft drinks, gaseous, cocoa milk, iced tea, sodas, sports drinks, energy drinks, oranges, lemons, kiwis, grapefruits, apples, peaches, and fruit yogurts was statistically significantly higher in students with erosion (p <0.05). There was no statistically significant relationship between students’ sex, systemic disease, premature birth and low birth weight, exercise activity level, socioeconomic status, parental education level, and oral hygiene habits with erosion (p >0.05).

**Conclusion:**

Although erosive lesions were limited to the enamel, the prevalence of erosion was high. Erosion was statistically significantly associated with older age, elevated BMI, consumption of certain beverages, and fruit.

Dental erosion is a chronic, localised, painless, progressive, and irreversible loss of dental hard tissues. In dental erosion lesions, hard tissues of teeth are chemically destroyed by acids they are exposed to without the involvement of bacteria.^29^

Dental erosion can affect both primary teeth and permanent teeth. The formation of erosions in permanent teeth is inevitable if the source of erosion in primary teeth is not determined, and if necessary precautions are not taken. Additionally, due to the wide width of pulps in young permanent teeth, dental erosion-induced pulp inflammation and pulp exposure may occur.^28^ For such reasons, early diagnosis and preventive measures of dental erosion in children are important to prevent permanent teeth from being affected by erosion.

Due to a change in lifestyle and dietary habits nowadays around the world, the consumption of acidic foods and beverages has increased, which has in turn increased the prevalence of erosion.^23^ Increased prevalence of dental erosion has led to widespread research on this issue.^14^

In Turkey, there is currently only limited published research on the prevalence of dental erosion, aetiologic factors leading to erosion, and erosive potentials of consumed beverages and foods.^10^,^11^,^42^ Moreover, dental erosion studies conducted worldwide do not fully reflect the conditions specific to Turkey. More research is necessary on potential aetiologic factors of dental erosion, the frequency of consumption of acidic foods and beverages, people’s consumption habits, and the prevalence of erosion.

Therefore, this study aimed to determine the prevalence of dental erosion in the teeth of children aged 7–14 years, and to investigate potential factors leading to erosion. For aetiologic factors of dental erosion to be determined, we aimed to find the percentage of body mass index (BMI) calculated using children’s height and weight, sex, age, systemic diseases (asthma, reflux, etc.), as well as birth weight, duration of gestation, beverage consumption habits, fruit consumption habits, sports habits, and oral hygiene habits. We aimed to reveal the relationship between these parameters and dental erosion, and thus to contribute to protective/preventive practices.

## Materials and Methods

### Obtaining Ethics Committee Approval and Required Permissions

The ethics committee report required for our study was obtained from the Clinical Research Ethics Committee of Sivas Cumhuriyet University (ID: 2017-09/03). The official permissions required for the study were obtained from the Research Planning Board of the Sivas Provincial Directorate of National Education.

### Sample Selection and Sample Size Calculation

This study was a cross-sectional analysis of children aged 7–14 years, who were studying in different primary and secondary school schools in the provincial centre of Sivas, selected through the convenience sampling method.

Out of 44,024 students aged 7–14 years in the provincial centre of Sivas, a total of 473 participants (209 primary school students; 264 secondary school students) were included in the study using the following formula (α = 0.05, d = ± 0.05, p = 0.30, q = 0.70, t = 1.96 / n = [N.t^2^.p.q] / [(N-1).d^2^ + t^2^.p.q]).

Eleven schools – six primary schools and five secondary schools – representing 20% of the schools, were selected using the cluster sampling method. We determined how many students would be taken from each school by using the proportional selection method in the stratified sampling method.

Individuals who were receiving orthodontic treatment, those with neurological/psychological problems, individuals using dental prostheses, individuals with communication problems, and individuals who had previously been treated for dental erosion were not included in the study.

### Calibration of the Surveyor

Measurements were performed by the researcher ET during dental examinations. Calibration of the surveyor was ensured by an expert pedodontist (AK) who had experience in dental erosion in the Department of Pediatric Dentistry, Faculty of Dentistry, Sivas Cumhuriyet University. The calibration of the surveyor was first carried out on a photograph before the start of the study, and subsequently on 50 children aged 7–14 who presented to the paediatric dentistry clinic.

### Distribution of the Informed Consent Forms

The schools where intraoral examinations would be performed were visited one day prior to the examination days. In cooperation with the school administration, each student was given an informed consent form and a parental questionnaire form (Appendix 1) – inquiring socioeconomic and educational levels of the family, and the weight, height, duration of gestation, birth weight, and systemic diseases of the student – to be sent to the family of the student. On the visit day of the examinations, the children who brought a signed consent form and a filled parental questionnaire form from their legal guardian were included in the study.

### Conducting Intraoral Examinations

The study was conducted between October 2018 and December 2018. The children were orally examined in the school building in a room consisting of a table and a chair in the students’ own classes. Examination sets (each consisting of a sterilised mouth mirror, a probe, and a dental tweezer), disposable cotton rolls, gloves, face masks, and an examination light pen (Varta Led Pen Light, Dischingen, Germany) held by the researcher were used during the examinations. Under the examination pen light, all external surfaces of permanent teeth were evaluated using cotton rolls and were assessed using the O’Sullivan index^41^ (Table 1) for dental erosion. The findings were recorded on the examination form. In our study, only the erosion of permanent teeth was evaluated, without the inclusion of primary teeth.

**Table 1:** O’Sullivan (2000) index^61^

Site of erosion on each tooth represented by an alphabet	
Code A	Labial only
Code B	Palatal only
Code C	Incisal/occlusal only
Code D	Labial and incisal/occlusal
Code E	Palatal and incisal/occlusal
Code F	Multisurface
Grade of severity denoted by a number (worst score for any individual tooth recorded)	
Code 0	Normal enamel
Code 1	Matt appearance of the enamel surface with no loss of contour
Code 2	Loss of enamel only
Code 3	Loss of enamel with exposure of dentin (ADJ visible)
Code 4	Loss of enamel and dentine beyond ADJ
Code 5	Loss of enamel and dentine with exposure of pulp
Code 9	Unable to assess (eg, tooth crowned or large restoration)
Area of surface affected by erosion (denoted by a +/− sign)	
Code −	Less than half of the surface affected
Code +	More than half of the surface affected

After completion of the examinations, the students received oral hygiene training and advice on dietary regulation and protection from dental erosion. Dental treatment needs of the children included in the study were determined and reported to their parents in writing after intraoral examinations of the children were performed.

### Completion of Questionnaires

Before their intraoral examinations, the children were asked to fill out a questionnaire (Appendix 2) that questioned their food consumption and behavioural habits, oral hygiene habits, and sports activities. While students from the older age group filled out the questionnaire form individually, the researcher assisted the younger students to fill out the questionnaires.

### Calculation of Body Mass Index (BMI)

After the children’s current heights and weights were measured, their body mass indices were calculated by using the formula proposed by World Health Organization (WHO): [BMI = weight in kilograms (kg)/height in meters (m)^2^].^50^ The BMIs were then placed on age- and sex-specific percentile curve charts specifically defined for the age range of 5–19 by WHO, and the children were classified based on their percentile values and ages.^50^

### Statistical Analysis

The obtained data were analysed using the SPSS software program (version 24.0; SPSS, Chicago, IL, USA). The conformity of the data to normal distribution was tested by using the Kolmogorov-Simirnov test. Independent sample t-tests were used to evaluate the relationship between dental erosion and the amount of beverage and fruit consumption. Tukey tests were used to quantify the relationship between mean age and dental erosion. The data obtained through counting were analysed using chi-square tests in a two-by-two multiway layout (the relationship between dental erosion, and sociodemographic data, general health status, sports habits, fruit and beverage consumption habits, and oral hygiene habits). Logistic regression analyses were carried out to determine risk factors for dental erosion. Binary logistic regression analysis was used to determine the factors affecting the development of dental erosion. Our data are shown in the tables as arithmetic means, standard deviations, and numbers and percentages of individuals. The percentage of error was considered to be 0.05.

## Results

### Dental Erosion Scores and Teeth with Erosion

Dental erosion was detected in 103 of the 473 students (21.8%). A total of 312 permanent teeth of the 103 students who were found to have dental erosion were affected by erosion (Table 2). The most frequently affected teeth from dental erosion were the teeth 21, 11, 22, and 12, in the order given. The most common dental erosion code was A1 (–) and was present in 47.4% of the teeth. The A1 (–) code was used only when the erosion was seen on the buccal surface, on the enamel, and on less than half of the tooth tissue. A2 (–), A1 (+), and C1 (–) codes followed the A1 (–) score in the order given (Table 2). The tooth surface that was most affected by erosion was the labial/buccal (code A) surface, and was observed in 67.5% of the teeth (Table 2). Code 1 in the index that was used refers to the matte appearance only in the enamel, without any loss of contour. Code 1 was detected in 78.2% of teeth affected by dental erosion (Table 2). When the surface areas affected by erosion were evaluated using the classification of dental erosions, code (–) erosion lesions were detected in 82.1% of the 312 teeth, in which less than half of the surface area was affected, while code + erosion lesions were detected in 17.9% of the teeth, in which more than half of the surface area was affected (Table 2).

**Table 2:** Determined dental erosion scores

Code	N	%	Site of erosion
A1 (–)	148	47.4	Labial or buccal only (67.5%)
A1 (+)	24	7.7	
A2 (–)	35	11.2	
A2 (+)	2	0.6	
A3 (–)	2	0.6	
B1 (–)	4	1.3	Lingual or palatal only (1.3%)
C1 (–)	22	7.1	Occlusal or incisal only (13.6%)
C1(+)	8	2.6	
C2 (–)	7	2.3	
C2 (+)	2	0.6	
C3 (–)	3	1.0	
D1 (–)	19	6.1	Labial and incisal/occlusal (15.0%)
D1 (+)	14	4.5	
D2 (–)	12	3.8	
D2 (+)	2	0.6	
F1 (–)	4	1.3	Multisurface (2.6%)
F1 (+)	1	0.3	
F2 (+)	3	1.0	
Total	312	100.0	
Code	N	%	Grade of severity of erosion
Code 1	244	78.2	Matt appearance of the enamel surface
Code 2	63	20.2	Loss of enamel only
Code 3	5	1.6	Loss of enamel with exposure of dentin
Total	312	100.0	
Code	N	%	Area of surface affected by erosion
Code –	256	82.1	Less than half of the surface affected
Code +	56	17.9	More than half of the surface affected
Total	312	100.0	

### The Relationship Between Dental Erosion, Sex, Age, Socioeconomic Status of Families, Education Level of Parents, Systemic Diseases, Percentage of BMI, Birth Time, and Birth Weight

Although there was no statistically significant relationship between dental erosion and sex (p = 0.157), the rate of erosion in males (24.6%) was numerically higher than in females (19.2%) (Table 3). No statistically significant relationship was observed between the age groups and dental erosion of the age groups of the children involved in the study was addressed separately (p = 0.078) (Table 3). However, the mean age of the students with dental erosion was statistically significantly higher versus students who did not have erosion (p = 0.001) (Table 4). There was no statistically significant relationship between dental erosion and socioeconomic status of families (p = 0.091), parental educational levels (mother and father) (p = 0.796, p = 0.930, respectively), presence of systemic disease (p = 0.158), premature birth or low birth weight (p >0.05) (Table 3). When the relationship between dental erosion and BMI was examined, dental erosion was statistically significantly more prevalent among overweight children (p = 0.019) (Table 3).

**Table 3:** Association between tooth erosion and sociodemographic characteristics, general health, sport habits, drink and food consumption habits, and oral hygiene habits

Variables	Erosion p resent n/(%)	Erosion absent n/(%)	Total n	
Sociodemographic characteristics				
Sex				
Female	47 (19.2)	198 (80.8)	245	X^2^ = 2.00
Male	56 (24.6)	172 (75.4)	228	p = 0.157
Age				
7	4 (8.5)	43 (91.5)	47	X^2^ = 12.75
8	15 (19.5)	62 (80.5)	77	p = 0.078
9	14 (17.5)	66 (82.5)	80	
10	13 (19.7)	53 (80.3)	66	
11	14 (27.5)	37 (72.5)	51	
12	18 (24.0)	57 (76.0)	75	
13	18 (31.0)	40 (69.0)	58	
14	7 (36.8)	12 (63.2)	19	
Socioeconomic status				
Low (<1600 TL)	38 (19.3)	159 (80.7)	197	X^2^ = 4.78
Moderate (1600–3200 TL)	47 (27.2)	126 (72.8)	173	p = 0.091
High (>3200 TL)	18 (17.5)	85 (82.5)	103	
Education level of father				
Primary school	24 (22.9)	81 (77.1)	105	X^2^ = 0.45
Secondary school	20 (20.8)	76 (79.2)	96	p = 0.930
High school	43 (22.6)	147 (77.4)	190	
University	16 (19.5)	66 (80.5)	82	
Education level of mother				
Primary school	41 (20.7)	157 (79.3)	198	X^2^ = 1.02
Secondary school	27 (20.3)	106 (79.7)	133	p = 0.796
High school	23 (24.2)	72 (75.8)	95	
University	12 (25.5)	35 (74.5)	47	
General health				
Systemic disease				
Present	18 (29.0)	44 (71.0)	62	X^2^ = 2.20
Absent	85 (20.7)	326 (79.3)	411	p = 0.158
BMI				
Thinness	4 (15.4)a	22 (84.6)	26	X^2^ = 7.97
Normal	59 (18.7)ab	256 (81.3)	315	p = 0.019*
Overweight	40 (30.3)b	92 (69.7)	132	
Duration of gestation				
Pre-term (<37 week)	10 (20.0)	40 (80.0)	50	X^2^ = 0.74
Term (37–41 week)	92 (22.2)	322 (77.8)	414	p = 0.690
Post-term (≥42 week)	1 (11.1)	8 (88.9)	9	
Birth weight				
Low (<2500 g)	11 (24.4)	34 (75.6)	45	X^2^ = 0.39
Normal (2500–4000 g)	86 (21.3)	318 (78.7)	404	p = 0.822
High (>4000 g)	6 (25.0)	18 (75.0)	24	
Sports habits				
Swimming in the pool				
Yes	50 (18.7)	217 (81.3)	267	X^2^ = 3.34
No	53 (25.7)	153 (74.3)	206	p = 0.067
Regular sporting habits				
Yes	26 (27.7)	68 (72.3)	94	X^2^ = 2.38
No	77 (20.3)	302 (79.7)	379	p = 0.123
Consumption habits				
Beverage consumption habits				
Drinks with straw	10 (8.3)a	110 (91.7)	120	X^2^ = 45.21
Drinks slowly with glass	66 (26.8)b	180 (73.2)	246	p = 0,001*
Drinks quickly with glass	14 (15.7)a	75 (84.3)	89	
Keeping drinks in the mouth Gargling with drinks	13 (72.2)c	5 (27.8)	18	
Beverage consumption time				
With meals	38 (18.7) ^a^	165 (81.3)	203	X^2^ = 22.68
Between mealtimes	5 (14.7) ^a^	29 (85.3)	34	p = 0.001*
Before bedtime	7 (87.5) ^b^	1 (12.5)	8	
Irregular consumption	53 (23.2) ^a^	175 (76.8)	228	
Fruit consumption habits				
Bite	93 (20.4) ^a^	362 (79.6)	455	X^2^ = 12.53
Suck	10 (55.6) ^b^	8 (44.4)	18	p = 0.001*
Oral hygiene habits				
Previous dentist visits				
Yes	73 (21.0)	275 (79.0)	348	X^2^ = 0.49
No	30 (24.0)	95 (76.0)	125	p = 0.482
Visiting dentists regularly				
1 time per 6 months	12 (34.3)	23 (65.7)	35	X^2^ = 4.64
1 time per a year	15 (26.3)	42 (73.7)	57	p = 0.098
When there is a pain	76 (19.9)	305 (80.1)	381	
Frequency of toothbrushing				
Less than 1 time per a week	15 (26.8)	41 (73.2)	56	X^2^ = 4.48
1 time per 2 or 3 days	17 (23.3)	56 (76.7)	73	p = 0.345
1 time per a day	34 (25.8)	98 (74.2)	132	
2 times per a day	30 (17.5)	141 (82.5)	171	
More than 3 times per a day	7 (17.1)	34 (82.9)	41	
Technique of toothbrushing				
From right to left	25 (19.8)	101 (80.2)	126	X^2^ = 6.76
Up and down	10 (12.7)	69 (87.3)	79	p = 0.080
Technique of toothbrushing				
Circular	9 (20.9)	34 (79.1)	43	
Mixed	59 (26.2)	166 (73.8)	225	
Timing of toothbrushing				
After meals	22 (19.8)	89 (80.2)	111	X^2^ = 4.40
Sometimes after meals	15 (20.0)	60 (80.0)	75	p = 0.353
Before bedtime at night	34 (27.9)	88 (72.1)	122	
Before at breakfast	7 (25.9)	20 (74.1)	27	
After breakfast – before bedtime	25 (18.1)	113 (81.9)	138	
Toothbrush replacement frequency				
3 months	47 (24.1)	148 (75.9)	195	X^2^ = 3.97
6 months	30 (25.0)	90 (75.0)	120	p = 0.137
12 months	26 (16.5)	132 (83.5)	158	
Mouthwash use				
Yes	7 (25.0)	21 (75.0)	28	X^2^ = 0.08
No	96 (21.6)	349 (78.4)	445	p = 0.670
Dental floss use				
Yes	7 (43.7)	9 (56.3)	16	X^2^ = 4.69
No	96 (21.0)	361 (79.0)	457	p = 0.030*

† Different small letters indicate statistical significance within each column; ‡ <0.05 statistically significant.

**Table 4:** Association between tooth erosion and mean age

	n	Mean age	Std. deviation	
Erosion present	103	10.73	2.01	t = 3.27
Erosion absent	370	10.00	2.02	p = 0.001*

† p <0.05 statistically significant

### The Relationship Between Dental Erosion and Swimming/Sporting Habits

When the students were categorised into two groups as swimmers or non-swimmers, regardless of the swimming habits, the rates of erosion among the students who were swimming were higher than among those who had never swam, although the difference was not statistically significant (p = 0.067) (Table 3). Similarly, there was no statistically significant relationship between dental erosion and regular sporting habits of the students (p = 0.123) (Table 3).

### The Relationship Between Dental Erosion and Beverage Consumption

Individuals with erosion consumed significantly more freshly squeezed orange juice, powdered drinks mixed with water, fruit juice, drinks with cola, orange soft drinks, gaseous, cocoa milk, ice tea, sodas, fruit sodas, sports drinks, and energy drinks versus individuals without erosion (p <0.05) (Table 5). Freshly squeezed orange juice was found to increase the likelihood of causing erosion by 2.8 times; drinks with cola by 3.6 times; gaseous drinks by 2.1 times; and energy drinks by 10.1 times (Table 6). Statistically significantly higher rates of dental erosion were found among students consuming drinks slowly with a glass, keeping drinks in the mouth, gargling with drinks, and consuming drinks before bedtime in the evening (p <0.05) (Table 3).

**Table 5:** Association between tooth erosion and dietary habits

Variables	Erosion	Mean	Median	Min.	Max.	p
Deitary habits						
Beverage consumption						
Milk	Present	1.15	1.00	0	5	0.583
	Absent	1.20	1.00	0	5	
Ayran	Present	1.52	1.00	0	5	0.582
	Absent	1.49	1.00	0	4	
Freshly squeezed orange juice	Present	0.93	0.00	0	5	0.001*
	Absent	0.42	0.00	0	3	
Powdered drinks mixed with water	Present	0.54	0.00	0	6	0.001*
	Absent	0.14	0.00	0	3	
Fruit juice	Present	1.50	1.00	0	6	0.001*
	Absent	0.98	1.00	0	4	
Tea	Present	1.16	1.00	0	5	0.583
	Absent	1.20	1.00	0	5	
Coffee	Present	0.25	1.00	0	4	0.062
	Absent	0.22	0.00	0	4	
Cola	Present	1.49	1.00	0	9	0.001*
	Absent	0.37	0.00	0	3	
Orange soft drinks	Present	0.97	1.00	0	8	0.001*
	Absent	0.31	0.00	0	3	
Gaseous	Present	1.00	1.00	0	7	0.001*
	Absent	0.28	0.00	0	3	
Cocoa milk	Present	1.19	1.00	0	5	0.001*
	Absent	0.77	1.00	0	4	
Iced tea	Present	0.51	0.00	0	2	0.001*
	Absent	0.23	0.00	0	2	
Sodas	Present	0.49	0.00	0	4	0.001*
	Absent	0.12	0.00	0	3	
Fruit sodas	Present	0.77	1.00	0	3	0.001*
	Absent	0.25	0.00	0	3	
Sports drinks	Present	0.31	0.00	0	4	0.001*
	Absent	0.03	0.00	0	3	
Energy drinks	Present	0.27	0.00	0	4	0.001*
	Absent	0.01	0.00	0	1	
Fruit consumption						
Orange	Present	1.36	1.00	0	5	0.032*
	Absent	1.08	1.00	0	10	
Strawberry	Present	3.88	3.00	0	13	0.209
	Absent	3.50	2.00	0	20	
Grape	Present	1.68	1.00	0	7	0.187
	Absent	1.40	1.00	0	10	
Watermelon	Present	1.67	1.00	0	10	0.186
	Absent	1.49	1.00	0	10	
Lemon	Present	0.64	1.00	0	4	0.001*
	Absent	0.07	0.00	0	2	
Kiwi	Present	1.17	1.00	0	5	0.001*
	Absent	0.67	0.00	0	4	
Grapefruit	Present	0.27	0.00	0	3	0.001*
	Absent	0.04	0.00	0	1	
Banana	Present	1.52	1.00	0	3	0.062
	Absent	1.40	1.00	0	4	
Apple	Present	1.67	1.00	0	5	0.013*
	Absent	1.36	1.00	0	5	
Peach	Present	1.40	1.00	0	5	0.017*
	Absent	1.01	1.00	0	5	
Pear	Present	1.04	1.00	0	4	0.556
	Absent	1.11	1.00	0	5	
Fruit yogurt	Present	0.92	1.00	0	5	0.001*
	Absent	0.48	1.00	0	10	

† p <0.05 statistically significant

**Table 6:** Logistic regression analysis of variables associated with dental erosion

Variables	B	Std. deviation	p	EXP (B) OR	95% CI for EXP (B)	
					Lower	Upper
Beverage						
Freshly squeezed orange juice	1.04	0.25	0.001*	2.83	1.72	4.66
Powdered drinks mixed water	0.62	0.36	0.088	1.87	0.91	3.85
Fruit juice	0.28	0.22	0.190	1.33	0.86	2.05
Cola	1.29	0.29	0.001*	3.64	2.04	6.51
Orange soft drink	0.59	0.33	0.081	1.80	0.92	3.50
Gaseous	0.76	0.33	0.021*	2.14	1.12	4.08
Cocoa milk	0.14	0.24	0.543	1.15	0.72	1.86
Iced tea	0.13	0.38	0.719	1.14	0.54	2.43
Sodas	0.00	0.38	1.000	1.00	0.47	2.12
Fruit sodas	0.64	0.35	0.069	1.90	0.95	3.79
Sports drinks	0.80	0.70	0.252	2.22	0.56	8.78
Energy drinks	2.30	1.02	0.024*	10.06	1.34	75.27
**Fruit**						
Orange	0.37	0.22	0.098	1.44	0.93	2.24
Lemon	2.97	0.48	0.001*	19.60	7.64	50.29
Kiwi	0.43	0.23	0.060	1.54	0.98	2.42
Grapefruit	0.95	0.64	0.142	2.59	0.72	9.21
Apple	0.15	0.22	0.513	0.86	0.55	1.34
Peach	0.09	0.23	0.688	1.10	0.69	1.75

† Exp (B) = odds ratio (OR); CL: confidence interval; p <0.05 statistically significant.

### The Relationship Between Dental Erosion and Amounts Fruit Consumption

The consumption of oranges, lemons, kiwis, grapefruits, apples, and peaches was statistically significantly higher among those with erosion versus those without erosion (p <0.05) (Table 5). When the risks of causing dental erosion of the fruits which were found to be related to erosion were examined, lemon was found to increase the likelihood of causing dental erosion by 19.6 times (OR: 19.6 [95% CI: 7.64–50.29]) (Table 6). Although it was found that the other fruits were related to erosion at a statistically significant level, it was determined that they did not increase the risk of causing dental erosion, based on the risk analysis. Moreover, with the increase in the consumption of fruit yogurt, dental erosion was found to be increased at a statistically significant level (p = 0.001) (Table 5). When the relationship between fruit consumption habits and dental erosion was examined, it was found that there was statistically more dental erosion among those who consumed fruits by sucking (p = 0.001) (Table 3).

### The Relationship Between Dental Erosion and Dentist Visits and Oral Hygiene Habits

Dental erosion was found not to be statistically significantly related to previous dentist visits, visiting dentists regularly, frequency of toothbrushing, technique and timing of toothbrushing, toothbrush replacement frequency, or with mouthwash use (all p >0.05) (Table 3).

## Discussion

The evolving food and beverage industry has increased the presence of carbonated beverages and sodas in the market and has caused an increased consumption of these beverages, especially by children. It is believed that the consumption of these beverages increases the prevalence and severity of tooth wear (erosion), which is caused by exposure to acidic factors.^49^ Dental erosion is affected by chemical properties of food and beverages (chelation properties, calcium, phosphate, and fluoride content), behavioural characteristics of patients (eating habits, lifestyles, excessive acid consumption), and the biological composition of saliva and teeth (saliva secretion rate, buffer capacity of saliva, pellicle formation, and the anatomy of the hard and soft tissue of teeth).^32^ For dentists, the determination of the aetiologic factor is at the top of the preventive procedures. By the classification of the degree of dental erosion observed in an individual, the erosive factors that the individual has been exposed to, and similarly the protective factors, can be identified. This is crucial to the solution of the problem and the application of protective/preventive practices for future dental erosions.^51^

In our study, the prevalence of students with dental erosion in at least one tooth was found to be 21.8%, which aligns with other published findings.^10^,^16^,^48^ In studies involving a Turkish population, Öcal^42^ noted dental erosion in 25.9% of children aged 11–15 years (n = 576) and Çağlar et al^10^ noted erosion in 28.0% of children aged 11 years (n = 153). Considering epidemiological studies, the wide range in prevalence of dental erosion can be attributed to the presence of a great number of variables, such as number of people in the sample, inclusion criteria, age group, examined teeth, and the index used for the diagnosis of erosion.

When the dental erosion scores in our study were examined, the most common score, similar to that of the other studies, was A1 (–), the affected surface was often the buccal surface,^3^,^16^,^20^ erosion was often limited on the enamel,^25^,^26^,^39^,^44^,^48^, and often less than half of the surface was affected by erosion.^25^,^35^ (Table 2) In the study, it is estimated that external acids were mostly effective in the formation of dental erosion lesions.

Due to the cleansing of the maxillary incisors by saliva is less due to their distance to the opening of the major and minor salivary glands,^30^ we think that dental erosion was detected more frequently on the buccal surface of the maxillary incisors. In our study, the fact that the dental erosion lesions were often observed in less than half of the surface in the enamel without loss of contour suggests that the severity of erosion was low and that the students included in our study had been exposed to erosive agents for a short time or continuously at low levels.

The mean age of the students with dental erosion was found to be statistically significantly higher than that of the students with no dental erosion (Table 4). Similar to the study of Zhang et al^54^ and Salas et al^44^, the prevalence of dental erosion increased with age. This finding might be explained by the tendency of erosion to progress and the exposure to erosive factors for prolonged times.^12^

The living standards of individuals appear to be directly associated with their financial revenues, educational levels, cultural values, and ethnic identities.^38^ Although there are studies reporting a relationship between low or high socioeconomic level and dental erosion in the literature,^16^,^36^,^54^ there was no statistically significant relationship between socioeconomic levels of families and dental erosion in our study (Table 3).^3^,^6^,^44^ The differences that emerge in studies investigating the socioeconomic level and dental erosion relationship might be due to the differences between the ages of children being examined, and therefore, different etiologic and environmental factors. Considering that parental educational level affects the living conditions of families, children take their parents as examples for their behaviours. Similarly with other published studies,^4^,^5^,^55^ there was no statistically significant relationship between parental educational levels and dental erosion (Table 3).

Children’s excessive consumption of carbonated beverages leads to the emergence of health problems, such as obesity and the deterioration of oral health (increased tooth decay and dental erosion).^26^ Although there are studies indicating that there is no correlation between BMI and dental erosion,^3^,^18^ there are also studies stating that there is a positive correlation between these two factors.^19^,^47^ Of the people who participated in the study of Isaksson et al^19^ – who investigated the frequency, distribution, and severity of dental erosion and their relationship to lifestyle, oral health, and general health – 69% were found to be of normal weight, 18% were overweight, 7% were obese, and 6% were underweight. Dental erosion was found to increase as BMI increased. When the relationship between dental erosion and BMI was examined, it was observed that dental erosion was increased statistically significantly among the children who had a greater BMI value than that for their age (Table 3). We think that obesity increases with excessive amounts of consumption of carbonated beverages and may be associated with dental erosion.

Many factors arising from low birth weight and premature birth can affect children’s tooth development and oral-dental health. Moreover, in children with low birth weight, the risk of tooth decay may also be observed more frequently due to biological and socioeconomic factors.^8^ O’Connell et al^40^ evaluated the dental health of children with low birth weight, aged 4–8 years. They reported that they detected dental erosion in 20% of 45 children. In our study, the relationship of premature birth and low birth weight to erosion was examined, and consequently, no statistically significant relationship was found (Table 3). When the literature was reviewed for this issue, only a few studies could be found to investigate the relationship of premature birth and low birth weight to dental erosion.^40^,^42^

It has been reported that in individuals who exercise and/or play sports frequently, the risk of dental erosion is increased due to the excessive consumption of acid-containing sports drinks and/or energy drinks.^9^,^37^ It has been suggested that when swimming professionally (often in chlorinated pools), when the level of saturation of water is less than the level of saturation of teeth surfaces, the pool water, which is in contact with teeth in the oral cavity for a long time, can cause erosion on teeth surfaces.^7^ Dental erosion has been reported to be higher among those who play sports regularly and have habits of swimming in the pool.^17^ Çağlar et al examined the relationship between dental erosion and swimming regularly in two clinical trial studies in 2005^10^ and 2011^11^ in Turkey. They, however, did not find a statistically significant difference in terms of the prevalence of dental erosion among children who were regularly swimming in the pool and those who were not. When the students were categorised into two groups of swimmers or non-swimmers, regardless of the swimming habits, the rates of dental erosion were higher among the students who were swimming versus those who had never swam, although the difference was not statistically significant (Table 3). In the section where the students’ sports habits other than swimming were inquired, however, it was seen that only 19.9% of the students (94 students) played sports regularly, whereas 80.1% (379 students) did not regularly play sports. There was no statistically significant relationship in the probability of dental erosion among those who were playing sports regularly for at least 1 year compared to those who did not play sports regularly (Table 3).

The consumed acidic beverages and foods are believed to be one of the aetiologic factors of dental erosion.^26^,^48^ Many researchers have examined the effects of carbonated beverages on enamel in in vitro studies, and defined these as a risk factor for dental erosion.^15^,^24^ Although there are studies stating that there is no relationship between beverage consumption and dental erosion,^6^,^25^,^44^,^54^ studies stating that the consumption of carbonated beverages,^3^,^39^ juice,^54^ and sports drinks^37^,^46^ are risk factors for dental erosion support the results of our study (Table 5). Carbonic beverages contain acid. Ready-made fruit juice contains carbonic acid and phosphoric acid. Freshly squeezed fruit juice, sports, and energy drinks contain fruit acids (such as citric acid) and have low pH levels. That is why they are thought to carry the potential to cause erosion on the enamel surface. Although it has been stated in some studies that milk has a protective effect against dental erosion,^13^,^39^ it is surprising that in our study, more dental erosion was detected among those consuming cocoa beverages with milk, similar to the results of Al-Dlaigan et al.^2^ We believe that a relationship was identified between dental erosion and milk with cocoa in our study because children consuming milk with cocoa also consumed a lot of acidic beverages, so this result could be misleading.

When the coefficients of the risk that consumed beverages cause dental erosion are investigated, there are studies stating that there is no relationship between consumed beverages (fruit juice, carbonated beverages, sports drinks, coffee, milk, and tea) and dental erosion.^44^,^6^ However, Kumar et al^26^ found that the consumption of carbonated beverages increased the risk of dental erosion by 2.80 times. Additionally, Hamasha et al^17^ found that the risk of dental erosion was increased by 12 times among those who consumed sports drinks two-to-four times per week, by 14 times among those consuming them once a day, and by 29 times among those consuming them more than two times per day. In our study, when the risks of causing dental erosion of the drinks which were found to be related to erosion were examined, freshly squeezed orange juice was found to increase the likelihood of causing dental erosion by 2.8 times; drinks with cola, by 3.6 times; soft drinks, by 2.1 times; and energy drinks, by 10.1 times (Table 6).

As the consumption time and consumption habits of acidic beverages are considered risk factors for dental erosion, the ways of consumption were questioned in the questionnaire section of our study. When the students’ beverage consumption times were examined, dental erosion was found to be significantly higher in children who consumed drinks before bedtime (Table 3). During meal consumption, saliva stimulation increases due to chewing, which can reduce the erosive potential of beverages. It has been recommended to limit the consumption of acidic products and carbonated beverages to mealtimes. Moreover, it has been reported that the consumption of acidic beverages before bedtime is a risk factor for children, specifically in terms of erosion due to the physiological lack of saliva secretion at night.^52^ These findings can be explained by the reduction of the cleansing effect of saliva due to the reduction of saliva secretion at night, and thus, the fact that the acidic drinks that are drunk during the aforementioned time period cause a more erosive effect on the teeth.

In the questionnaire section of our study, the ways in which the students consumed drinks were examined, and those who drank drinks slowly with a glass and those who kept drinks in the mouth and gargled them were found to have significantly more dental erosion (Table 3). Johansson et al^22^ have determined that the presence of large-volume beverages in the mouth adversely affects the intraoral pH, compared to a smaller volume during the same time period. Moreover, Shellis et al^45^ have determined that the depth of erosion increases linearly with the duration of exposure to the beverage. These findings support the presence of more dental erosion among those who drink beverages slowly with a glass.

Citrus fruits are known to contain fruit acids such as citric acid, a factor that may cause dental erosion. In our study, similar to the results of Kumar et al^26^ and Hamasha et al^17^, it was found that lemon consumption, in particular, was a statistically significant risk factor for dental erosion (Table 6). Furthermore, significantly greater dental erosion was detected among those who frequently consumed fruits such as oranges, kiwis, grapefruits, apples, and peaches (Table 5). Studies linking citrus consumption with dental erosion support the results of our study.^3^,^17^,^26^ We believe that these fruits were found to be associated with dental erosion due to their citric acid content and low pH. Considering that unusual fruit consumption habits may be a risk factor in terms of dental erosion, the students’ fruit consumption habits were inquired in our questionnaire, and significantly more dental erosion was detected in those who consumed fruits by sucking (Table 3). There are studies^27^,^31^ indicating that certain fruits, such as lemons, apples, plums and peaches have low pH values, and when they are consumed by sucking they increase dental erosion significantly, which supports our findings. We think that fruit consumption by sucking may affect the formation of dental erosion more prominently due to the fact that acidic fruits are more in contact with teeth if they are kept in the mouth and teeth become exposed more to acidic environment for a longer period of time.

It can be seen that there are studies stating that there is a protective effect of yogurt against dental erosion due to Ca and PO_4_–3 in its composition.^34^,^43^ However, studies^21^,^33^ stating that there is an abrasive effect of yogurt on the enamel supports our result that more dental erosion was detected among the students who consumed more fruit yogurt (Table 5). We think that the acid regulators (citric acid) found in the content of fruit yogurt and lactic acids, which are naturally found in the yogurt culture, may be the factors responsible for the development of dental erosion.

Visiting a dentist is directly related to the creation of awareness about oral health. It is known that individuals conscious about oral health brush their teeth regularly, and therefore, succeed in plaque control. However, it has been stated that tooth surfaces of such persons are deprived of the protective power of dental plaque against acid attacks, due to which their sensitivity to dental erosion increases because of factors such as acidic products, abrasive effect of toothpastes, and strong toothbrushing.^1^ Although good oral hygiene is valuable in the prevention of periodontal diseases and tooth decay, it is noted that frequent toothbrushing can accelerate dental erosion.^53^

Similar to the results of our study, the majority of the studies in the literature are those that do not identify statistically significant relationships between dental erosion and the frequency of going to the dentist and oral hygiene habits (the toothbrushing technique, toothbrushing frequency, technique and time, toothbrush replacement frequency, and use of mouthwash).^3^,^6^,^17^,^18^,^26^,^36^,^39^ (Table 3) Although the majority of the students participating in the study had previously been to a dentist, they were not diagnosed with dental erosion. This may be attributed to the possibility that the dentists were not sufficiently focusing on dental erosion and not providing sufficient information to parents about erosion detection or protective measures. It is thought that although the majority of the students were found to brush their teeth two times per day – after breakfast and before bedtime – other factors such as the force applied during brushing and the abrasive effect of the toothpaste used may be prominent in the formation of erosion, which is why no relationship could be found between oral hygiene habits and dental erosion.

### Study Limitations

We believe that in epidemiological studies, where questionnaires are used as data collection instruments in schools, there may be differences between the answers to the questions given by the child and the parent. To control the reliability of the child’s answers in the school alone, it is necessary that this questionnaire be filled under parental supervision. A limitation of our study was that a direct communication with the parent was not possible.

## Conclusion

When epidemiological studies are examined, it is seen that the prevalence of dental erosion and the factors associated with dental erosion vary, which may be attributed to the presence of many variables such as the index used for the diagnosis of erosion, number of people in the sample, criteria for inclusion in the study, age group, examined teeth, socioeconomic level, and geographical conditions.

Food and beverage consumption habits may vary between countries worldwide, and even between different regions within country. For these reasons, it is necessary to consider the eating and drinking habits of the countries when investigating the risk factors for dental erosion. In this context, it is necessary for dentists to collect a detailed medical and social history to have information about daily activity, personal nutrition, and oral hygiene habits of their patients. Owing to the increasing prevalence of dental erosion, dentists need to understand the aetiologic factors of erosion, so they may counsel their patients. Providing information on reducing consumption of acidic beverages and foods, maintaining balanced nutrition, and good oral hygiene will help patients take measures to minimise risk.

## Appendix 1 Parents’ Questionnaire

**Children’s name, surname:**

**Children’s age:**

**Children’s height:**

**Children’s weight:**

**Children’s systemic disease:** Present ( ) Absent ( )

Gastrointestinal diseases ( ) Asthma ( ) Cardiovascular diseases ( )

Diabetes mellitus ( ) Others ( ) Enter the name.....

**Duration of gestation:** <37 week ( ) 37–41 week ( ) ≥42 week ( )

**Birth weight:** <2500 g ( ) 2500–4000 g ( ) >4000 g ( )

**Education level of father:** Primary school ( ) Secondary school ( ) High school ( ) University ( )

**Education level of mother:** Primary school ( ) Secondary school ( ) High school ( ) University ( )

**Socioeconomic status:** <1600TL ( ) 1600–3200TL ( ) >3200TL ( )

## Appendix 2 Childrens Questionnaire

### Dietary habits

**Beverage consumption on the basis of cups**

**Table:**

Milk	Fruit juice	Orange soft drinks	Sodas
Ayran	Tea	Gaseous	Fruit sodas
Freshly squeezed orange juice	Coffee	Cocoa milk	Sports drinks
Powdered drink mixed with water	Cola	Iced tea	Energy drinks

**Fruit consumption**

**Table:**

Orange	Watermelon	Grapefruit	Peach
Strawberry	Lemon	Banana	Pear
Grape	Kiwi	Apple	Yogurt with fruit

**Beverage consumption habits:**

Keeping drinks in the mouth, Gargling with drinks ( )

Drinks with straw ( ) Drinks slowly with glass ( ) Drinks quickly with glass ( )

**Beverage consumption time:**

With meals ( ) Between mealtimes ( ) Before bedtime ( ) Irregular consumption ( )

**Fruit consumption habits:** Bite ( ) Suck ( )

**Sports Habits**

**Swimming in the pool:** Yes ( ) No ( )

Never swum ( ) Swam regularly ( ) Swam only in the summer for less than one month ( )

**Regular sporting habits:** Yes ( ) No ( )

**Oral Hygiene Habits**

**Previous dentist visits:** Yes ( ) No ( )

**Visiting dentists regularly:**

1 time per 6 months ( ) 1 time per a year ( ) When there is a pain ( )

**Frequency of toothbrushing:**

Less than 1 time per a week ( ) 1 time per 2 or 3 days ( ) 1 time per a day ( )

2 time per a day ( ) More than 3 times per a day ( )

**Technique of toothbrushing:**

From right to left ( ) Up and down ( ) Circular ( ) Mixed ( )

**Timing of toothbrushing:**

After meals ( ) Sometimes after meals ( ) Before bedtime at night ( )

Before at breakfast ( ) After breakfast – before bedtime ( )

**Toothbrush replacement frequency:**

3 months ( ) 6 months ( ) 12 months ( )

**Mouthwash use:** Yes ( ) No ( )

**Dental floss use:** Yes ( ) No ( )
